# Complete Genome Sequence of Bacillus coagulans BC01, a Promising Human Probiotic Strain Isolated from Thick Broad Bean Sauce

**DOI:** 10.1128/MRA.00392-21

**Published:** 2021-05-13

**Authors:** Yang Yu, Xueping Yu, Jialiang Ouyang, Xin Ma

**Affiliations:** a Thankcome Biological Science and Technology Co., Ltd., Suzhou, China; b State Key Laboratory of Bioreactor Engineering, East China University of Science and Technology, Shanghai, China; University of Rochester School of Medicine and Dentistry

## Abstract

We report the whole-genome sequence of a promising human probiotic, Bacillus coagulans BC01, isolated from thick broad bean sauce. *B. coagulans* BC01 is widely used in China, where it is considered a treatment for diarrhea, constipation, and allergies and an immunity booster. The complete genome sequence of *B. coagulans* BC01 will help future research to provide more molecular information about its features and safety.

## Abstract (precis)

We report the whole-genome sequence of a promising human probiotic, Bacillus coagulans BC01, isolated from thick broad-bean sauce. *B. coagulans* BC01 is widely used in China, where it is considered a treatment for diarrhea, constipation, and allergies and an immunity booster. The complete genome sequence of *B. coagulans* BC01 will help future research to provide more molecular information about its features and safety.

## ANNOUNCEMENT

Probiotics have been demonstrated to have a significant impact on human health. Among all probiotics, the bacterial species belonging to the genus *Bacillus* are indeed remarkable for their high tolerance to gastric acids and bile salts (1). They have been reported to play a significant role in preventing irritable bowel syndrome infection (2), enhancing immunity (3), inhibiting the growth of pathogenic bacteria (4), and treating diarrheal diseases in the human body (5). Bacillus coagulans was described at the Iowa Agricultural Experiment Station in 1915 by Sarles and Hammer (6). We believe that *B. coagulans* BC01 holds great promise for the probiotic enterprise, and it would be highly useful for researchers to further explore the functional mechanisms of probiotics related to human health.

We isolated *B. coagulans* BC01 in 2015 from homemade thick broad bean sauce in Sichuan Province, China. One gram of the sample was serially diluted and spread over MRS medium agar, followed by incubation at 42°C for 2 days. One single white colony growing on the plates after 2 days’ incubation was identified as Bacillus coagulans (99.73% identity to the 16S rRNA gene of *B. coagulans* LBSC [GenBank accession number CP022701.1]) based on 16S rRNA gene sequencing. The primers used for amplification were 27f (5′-AGAGTTTGATCCTGGCTCAG-3′) and 1492r (5′-GGTTACCTTGTTACGACTT-3′). The NCBI BLAST database (https://blast.ncbi.nlm.nih.gov/Blast.cgi) was used for determination of the 16S rRNA identity. *B. coagulans* BC01 has been archived in the China Center for Type Culture Collection since 2017 and was numbered CCTCC number M2017813.

The DNA of BC01 was extracted from the original isolation in 2015; a single colony was inoculated in MRS medium and cultured at 42°C for 48 h (7). High-quality genomic DNA was extracted using a modified cetyltrimethylammonium bromide (CTAB) method (8, 9). The quality and quantity of the extracted DNA were examined using a NanoDrop 2000 spectrophotometer (Thermo Fisher Scientific, Waltham, MA, USA), a Qubit double-stranded DNA (dsDNA) high-sensitivity (HS) assay kit on a Qubit 3.0 fluorometer (Life Technologies, Carlsbad, CA, USA), and electrophoresis on 0.8% agarose gel, respectively. Genomic DNA was fragmented using g-TUBEs (Covaris) and then end repaired to prepare SMRTbell DNA template libraries (with a fragment size of >10 kb selected using the BluePippin system) according to the manufacturer’s instructions (Pacific Biosciences). The library quality was analyzed by Qubit, and the average fragment size was estimated using an Agilent (Santa Clara, CA, USA) 2100 Bioanalyzer. Single-molecule real-time (SMRT) sequencing was performed using a Pacific Biosciences Sequel II sequencer (Frasergen Bioinformatics Co., Ltd., Wuhan, China) and standard protocols (30-h movie) using Sequel II binding kit 2.0 chemistry. The raw sequencing reads (also called the polymerase reads) generated from the PacBio platform were processed using SMRTLink v8.0 with the parameter minLength set to 50 and all other parameters kept at default (https://www.pacb.com/support/software-downloads/). This software suite removes the hairpin adapter sequences from the polymerase reads to produce subreads. Subreads with a length of <50 bp were filtered out. Error correction of the reads was performed as part of the Canu v2.0 assembly process. A single SMRT cell produced a total of 2.25 Gb in 236,012 subreads (mean length, 9,527 bp), and the *N*_50_ value of 11,344 bp was used for *de novo* assembly with Canu software (10, 11). The average genome coverage was 526-fold. Genome annotation was completed by submission to the NCBI Prokaryotic Genome Automatic Annotation Pipeline (PGAP) (12).

The genome was assembled using Canu, and the results showed that it has a circularized structure. Canu v2.0 was used with the parameters “-pacbio genomeSize = 3.6m rawErrorRate = 0.300 errorRate = 0.045 minReadLength = 1000 minOverlapLength = 500.” Canu detects and annotates the final assembly as circular when the best overlap graph (BOG) is circular. As an alternative method, we confirmed genome circularization by identifying repeated sequences at the two ends using BLASTN v2.7.1. Lastly, we aligned the assembly to a reference genome of the same species (GenBank accession number CP017888.1) and determined its circular structure based on the whole-genome alignment. The assembly sequence was reordered such that the beginning of the assembly starts with the *dnaA* gene, resulting in one circular chromosome with a single contig of 3,564,910 bp. The chromosomal contig showed an average G+C content of 46.23%. It was predicted to contain a total of 4,180 genes, 30 rRNAs, and 85 tRNAs. Furthermore, antibiotic resistance gene analysis performed by ResFinder software v4.0 (13) predicted the gene *maf*(A), associated with resistance to macrolide antibiotics. Default parameters were used for all software unless otherwise specified.

### Data availability.

The complete genome sequences of Bacillus coagulans BC01 are available from GenBank under the accession number CP064767. The raw sequence reads have been deposited in the NCBI Sequence Read Archive (SRA) under the BioProject accession number PRJNA673749 and the BioSample accession number SAMN16631050.
